# Intracranial abscess due to *Mycobacterium avium* complex in an immunocompetent host: a case report

**DOI:** 10.1186/s12879-015-1026-5

**Published:** 2015-07-23

**Authors:** Mudit Chowdhary, Umesh Narsinghani, Ritu A. Kumar

**Affiliations:** Department of Internal Medicine, Mercer University School of Medicine, 707 Pine Street, Macon, GA 31201 USA; Department of Pediatrics, Mercer University School of Medicine, Macon, GA USA; Department of Infectious Diseases, Mercer University School of Medicine, Macon, GA USA

**Keywords:** *Mycobacterium avium*, MAC, CNS, Brain abscess, Immunocompetent

## Abstract

**Background:**

*Mycobacterium avium* complex (MAC) is a ubiquitous pathogen, widely distributed in the environment including water, soil and animals. It is an uncommonly encountered clinical pathogen; primarily causing pulmonary infections in patients with underlying lung disease or disseminated disease in immunocompromised hosts. Sporadically, extra-pulmonary infections have been documented including involvement of the liver, spleen, skin, soft tissue and lymph nodes. Central nervous system (CNS) infections due to MAC are exceedingly rare and carry a poor prognosis. Additionally, such infections are largely reported in patients infected with HIV. Herein we report the first case of intracranial abscess due to MAC in an immunocompetent man with a normal CD4 count and negative HIV status.

**Case presentation:**

A previously healthy 40-year-old male presented to us with progressively worsening CNS symptoms. The patient’s presentation was uncharacteristic of MAC infection in immunocompetent hosts, as he developed subacute, progressive symptoms that included severe frontal headaches, left eyelid swelling, blurry vision, and diplopia, without any pulmonary or systemic manifestations. Neuroimaging revealed multiple ring-enhancing lesions, which required neurosurgical intervention. MAC was the only pathogen that grew from intraoperative tissue cultures. The patient was subsequently treated with a 12-month regimen consisting of Clarithromycin, Ethambutol, and Rifampin, with successful clinical resolution.

**Conclusion:**

Our findings indicate that it is important to consider rare infections such as MAC in immunocompetent patients, regardless of atypical symptoms. Despite the severity of this infection, with timely diagnosis effective treatment is available.

## Background

The most prevalent nontuberculous mycobacterium (NTM) capable of causing disease in humans is *Mycobacterium avium* complex (MAC) [1]. Organisms classified as MAC comprise of at least two related species namely *Mycobacterium avium* and *Mycobacterium avium-intracellulare*. These organisms have been recovered from water, soil, food and milk, domestic and wild animals, though they are readily cleared in most humans.

Infection with MAC can be acquired by ubiquitous environmental exposure. It most commonly causes pulmonary infections in patients with chronic lung diseases, and disseminated disease in the immunocompromised host [1, 2].

NTM infection, while an extremely rare cause for CNS infection, portends a significant mortality rate, ranging from 35-70 % [3]. Studies have shown that disseminated disease, previous neurosurgery, and trauma are leading factors for CNS infection [2]. The majority of cases involving MAC infection of the CNS are seen as opportunistic infections in patients with acquired immunodeficiency syndrome (AIDS) with a severely depressed CD4 count (<50 cells/μl) [4].

Here, we report the first case of brain abscess caused by MAC in an HIV-negative male without any active underlying immunodeficiency.

## Case presentation

A 40-year-old Caucasian male presented with a 6-week history of chronic unremitting frontal headaches, followed by left eyelid swelling, blurry vision, and diplopia. He subsequently developed nausea and vomiting. He denied any fever, nasal congestion, night sweats, or weight loss. His past medical history was significant for bipolar disorder with no other relevant findings. The patient was not using any current medications and he denied a history of immunosuppresive drugs. Moreover, his family history was only positive for hypertension, without any mention of autoimmune disease. On examination, the patient was afebrile, pulse was 69 per minute, respirations were 23 per minute, and blood pressure was 150/89 mmHg.

The physical exam was notable for exophthalmos of the left eye as well as a palpable mass on the left eyelid. Neurological exam revealed third, fourth, and sixth cranial nerve palsy in the left eye. The patient also displayed decreased visual acuity in the affected eye with associated early disc edema. The remainder of the physical exam was unremarkable. The patient’s complete and differential blood counts, serum chemistry and erythrocyte sedimentation rate were normal. Given the findings, the patient was referred for computed tomography (CT) to rule out a mass lesion.

CT of the orbit with contrast revealed two frontal lobe lesions, the largest of which appeared solid measuring 3.6 × 3.8 cm on coronal image. The lesion was seen eroding through the orbital roof, exerting significant mass effect on the superior rectus muscle, and causing deformity of the left globe. An additional ring-enhancing left frontal lesion was seen adjacent to the solid tumor measuring 2.7 × 1.9 × 2.7 cm.

**Fig. 1:**
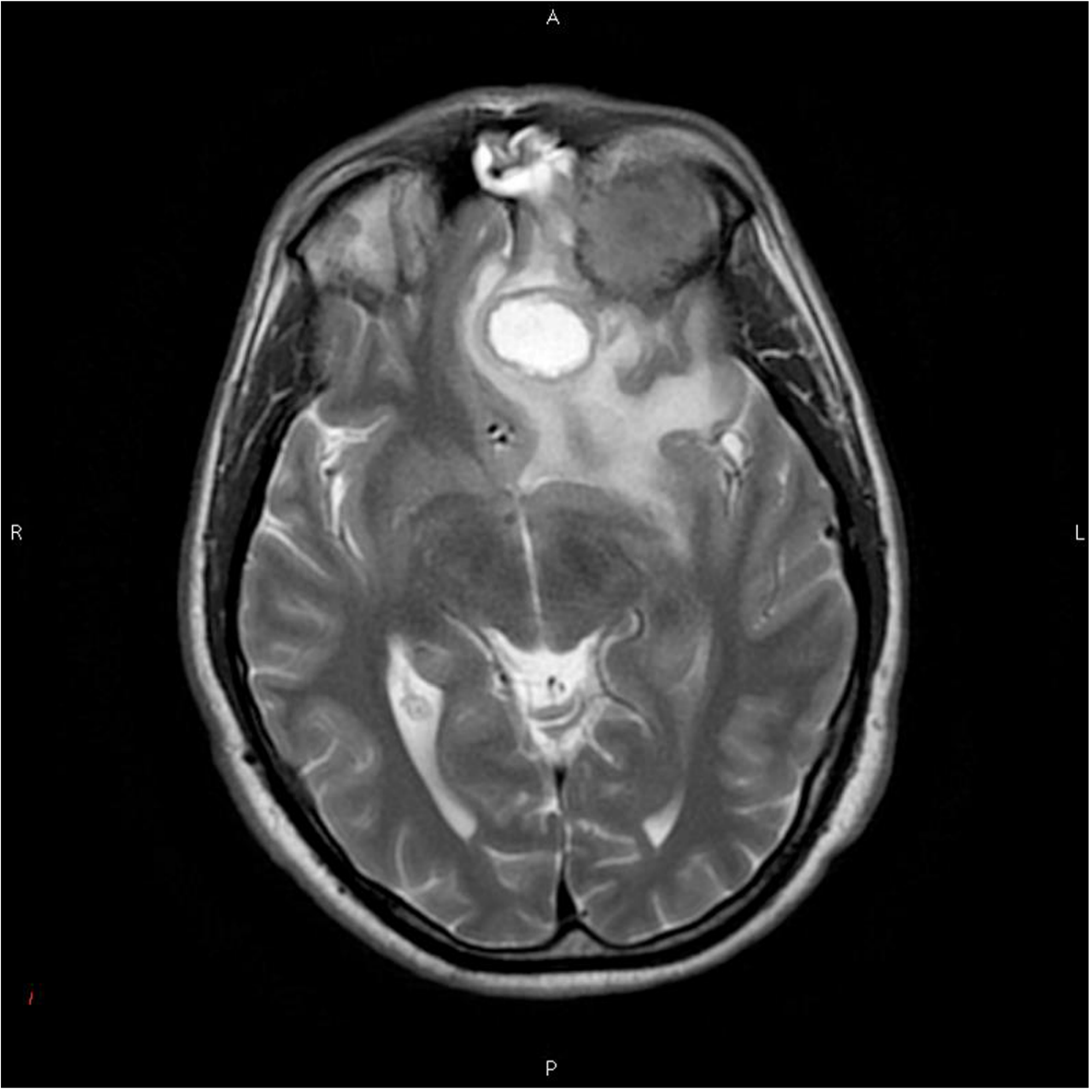
Axial T2 MRI showing a round hyperintense cystic area with surrounding vasogenic edema, and orbital involvement along with left-to-right midline shift due to mass effect

Subsequent magnetic resonance imaging (MRI) of the brain with and without contrast revealed a mass arising in the left orbital roof with destruction of the sphenoid bone. Posterior to the mass was a round hyperintense cystic area within the left inferior frontal cortex with surrounding severe vasogenic edema. Mass effect was visualized with midline shift from left to right of the septum pellucidum and subfalcine herniation (Fig. 1). The contrast enhanced study showed an extensive lytic mass measuring at least 3.4 × 2.2 cm in size. At the time, the etiology of the lesions was felt to be more likely neoplastic rather than infectious in the absence of any known immunocompromised state. Therefore, blood cultures were not obtained.

The patient consequently underwent a left frontal craniotomy for partial resection of the masses. Upon incision the large left frontal lobe lesion was full of purulent material from which multiple cultures were acquired. At this point, the differential favored intracranial abscess rather than neoplasm. Post-operative MRI noted that the previous ring-enhancing lesions were decreased in size. Additionally, the left retro-orbital hyperdense mass had resolved. The prior midline displacement was improved. Persistent vasogenic edema throughout the left frontal lobe was noted; however, the mass effect had resolved. Preliminary pathology from abscess resection revealed granulomatous inflammation and necrosis with positive acid-fast bacilli (AFB) cultures. Intraoperative bacterial, anaerobic and fungal cultures however were all negative. Routine blood cultures, AFB blood cultures and QuantiFERON® TB Gold test were also negative. The patient’s sputum culture however was positive for AFB. Additionally, the patient was tested for HIV following abscess resection with negative results. Moreover, the patient’s CD4 count was 515 cells/mm^3^ (32 %). The patient was started on an anti-TB regimen consisting of Rifampin, Isoniazid, Pyrazinamide, and Ethambutol.

CT of the thorax was performed to rule out a concomitant pulmonary infection. The study demonstrated calcified bilateral hilar lymph nodes, as well as multiple calcified mediastinal lymph nodes involving the prevascular, pretracheal, precarinal, right paratracheal, and subcarinal space, reflecting prior granulomatous disease or sarcoidosis. Results from endobronchial biopsy revealed noncaseating granulomas. Final tissue cultures from the brain lesions and sputum revealed MAC. The patient was started on a 12-month MAC treatment regimen consisting of Clarithromycin, Ethambutol, and Rifampin. The patient’s neurological symptoms, exopthalmous, diplopia and mass effect fully resolved over the following 6-months. The blurry vision persisted, but was corrected with glasses. Sputum AFB was negative at 7-months. Furthermore, follow-up MRI at 8-months was negative for intracranial abscess.

## Discussion

*Mycobacterium avium* complex typically affects the lungs, but other manifestations have been described including involvement of spleen, mesenteric lymph nodes, liver, and intestines [5, 6, 7]. MAC infections have been diagnosed in patients without predisposing conditions or immunosuppression, but they typically present as focal pulmonary or gastrointestinal diseases or occasionally in disseminated forms usually sparing the CNS [1, 8, 9]. Disseminated disease may complicate MAC pulmonary disease through local multiplication and entry into the bloodstream with seeding of other organs.

The clinical presentation of MAC lung disease is non-specific, highly variable and is influenced by whether the patient has pre-existing pulmonary disease. The most common clinical manifestations of disseminated MAC include intermittent or persistent fever, night sweats, weight loss, with additional symptoms of fatigue, malaise and anorexia. There has been no consistent immune deficiency identified to explain MAC infection in HIV-negative patients. However, apparent genetic defects of disseminated NTM infection have been associated with specific mutations in interferon (IFN)-γ and interleukin (IL)-12 synthesis and response pathways [10]. Additionally, specific non-immunocompromised groups have shown a predisposition for MAC pulmonary infection; reports show an increasing prevalence of bronchiectatic pulmonary MAC disease in elderly women without underlying risk factors [11].

Nevertheless, CNS infection due to MAC remains rare in patients with immunodeficiency, with even fewer case reports published on CNS infections due to MAC in immunocompetent patients. In 2011, a large-scale study was conducted to identify cases of CNS infections due to NTM. The authors identified only 15 out of 5960 (<1 %) patients with NTM CNS infection; 11 out of these 15 patients were HIV-seronegative. Among the patients, 1 had an infection due to *Mycobacterium kansasii*, 7 had infections due to *Mycobacterium abscessus,* and 1 presented with a mixed *M. abscessus-Mycobacterium fortuitum* infection. Only 2 cases were due to MAC (13 %) [3]. Furthermore, an extensive search in the English literature found only 8 additional cases with MAC brain abscess (Table 1): 7/8 patients were HIV-positive or presented with an underlying immunodeficiency [4, 9, 12–17]. Males were infected 75 % of the time and the median age was 38 years. Additionally, the median CD4 count was only 90 mm^3^ (range 2-215 mm^3^). The additional immunocompetent case diagnosis is unclear as the patient presented with risk factors for MAC infection. That case described a patient with chronic granulomatous meningitis who subsequently developed a secondary MAC brain abscess as a consequence of immunosuppresion due to prednisone therapy [12]. Moreover, the CD4 count was unknown. Our patient however, presented with a normal CD4 count, negative HIV status and without any existing co-morbidities.

**Table 1:** Summary of previous cases in the English literature

Case (Reference)	Age/Sex	CD4 mm^3^	HIV +/-	Site	Imaging	Comorbidity
1 [4]	42/F	14	+	Fronto-parietal	Single, ring enhancing mass, with mass effect	Leptomeningitis
2 [9]	52/M	175	-	Occipital cerebellum	Single, ring enhancing mass	Tuberculosis sarcoidosis
3 [11]	38/M	90	-	Frontal, parietal	Multiple masses, edema	Sarcoidosis
4 [12]	31/F	N/A	-	Temporal	Single mass, edema	MAC meningitis, prednisone therapy
5 [13]	35/M	215	+	Fronto-parietal	Single mass, edema	HAART, cryptococcal meningitis
6 [14]	40/M	31	+	Occipital	Single mass, edema	HAART, CMV, retinitis
7 [15]	33/M	2	+	Frontal, parietal, occipital	Multiple masses, ring enhancing	Disseminated MAC
8 [16]	36/M	170	+	Temporal, temporo-parietal	Multiple masses	HAART, Disseminated MAC, PCP, oropharyngeal candidiasis
Our case	40/M	515	-	Frontal	Single, ring enhancing mass, edema and mass effect, with invasion	-

*MAC* Mycobacterium avium complex, *HAART* Highly active antiretroviral therapy, *CMV* Cytolomegalovirus, *PCP Pneumocystis* pneumonia

Diagnosis of CNS MAC may be difficult and challenging given the rarity of the condition, the potential differential diagnosis of neoplasm as well as the varying presentations of MAC in the central nervous system [9, 12]. Patients presenting with focal neurologic symptoms and signs of headache, visual impairment, ophthalmoplegia, or papilledema warrant urgent evaluation. MRI is superior to CT in distinguishing suspected brain abscess, but culture of the specimen is the gold standard for accurate diagnosis [9, 18]. The specimen should be sent for Gram’s stain, aerobic, anaerobic, mycobacterial, and fungal cultures including histopathology.

Studies have failed to identify an optimal treatment regimen in MAC CNS infections, further complicating these cases. There is no relation between in vitro drug susceptibility and clinical response for agents other than the macrolides [19]. Clarithromycin is currently the only drug for which susceptibility testing is recommended, and is considered to be the mainstay of treatment in combination with other agents such as Ethambutol [19]. Other chemotherapeutic options for treatment of MAC include rifampin, rifabutin, aminoglycosides such as amikacin, and a flouroquinolone like moxifloxacin. The penetration of anti-NTM drugs to CNS infections is further complicated by the relatively impermeable blood-brain barrier (BBB). Ethambutol and the macrolides only reach sufficient CSF concentrations in the presence of meningeal inflammation [20]. Although rifampin shows greater efficacy in penetrating the BBB than the other drugs, it may be below the minimum inhibitory concentration level for some microbial strains [20, 21].

Guidelines from the Centers for Disease Control and Prevention, American Thoracic Society (ATS) and Infectious Diseases Society of America (IDSA) suggest initiating treatment with at least two drugs for disseminated MAC infection to prevent or delay the emergence of resistance [19]. Clarithromycin is the preferred first line agent with Ethambutol as the second recommended drug. Some clinicians may consider adding a third drug, either rifabutin or rifampin, in patients with disseminated MAC and HIV. Alternatively, azithromycin can be substituted for clarithromycin as it is efficacious and unlike clarithromycin, does not interact with rifabutin or rifampin in patients with AIDS [19]. In HIV patients, the United States Public Health Service and ATS/IDSA guidelines recommend minimum therapy for 12 months and 6 months of immune reconstitution [19]. With MAC pulmonary disease, treatment should be continued until sputum cultures are consecutively negative for at least one year.

## Conclusion

In summary, it is important to consider rare infections such as MAC in immunocompetent patients, regardless of atypical symptoms. We encourage clinicians to keep a high index of suspicion for this condition in order to prevent morbidity and mortality from disease progression. Furthermore, we stress the importance of obtaining tissue cultures in order to make a conclusive diagnosis. Combination drug therapy is essential to decrease the risk of resistance. Additionally, treatment with more than one agent is also associated with more rapid clearance of MAC from the bloodstream. Despite the severity of this infection, with timely diagnosis effective treatment is available.

## Consent

Written informed consent was obtained from the patient for publication of this Case report and any accompanying images. A copy of the written consent is available for review by the Editor of this journal.
